# Characteristics of the Extent and Onset of Osteonecrosis of the Femoral Head After Femoral Neck System (FNS) Fixation: A Minimum Two-Year Follow-Up Comparative Study with Cannulated Screws

**DOI:** 10.3390/jcm15166405

**Published:** 2026-08-19

**Authors:** Incheol Kook, Sihoon Choi, Soo-Young Jeong, Kyu Tae Hwang

**Affiliations:** 1 Department of Orthopedic Surgery, Cheongju TOP Hospital, 1 Gyoseo-ro, Sangdang-gu, Cheongju 28546, Republic of Korea; nathan0319@naver.com; 2 Department of Orthopaedic Surgery, Medi-in Hospital, 90 Geumneungyeok-ro, Paju 10913, Republic of Korea; sihoon812@gmail.com; 3 Department of Orthopedic Surgery, Sanggye Paik Hospital, 1342, Dongil-ro, Nowon-gu, Seoul 01757, Republic of Korea; jnjsywow@naver.com; 4 Department of Orthopaedic Surgery, Hanyang University Seoul Hospital, 222 Wangsimni-ro, Seongdong-gu, Seoul 04763, Republic of Korea

**Keywords:** femoral neck fractures, femur head necrosis, fracture fixation, internal, bone screws, postoperative complications

## Abstract

**Background/Objectives:** This study aimed to compare the incidence, extent, and time to diagnosis of osteonecrosis of the femoral head (ONFH) between Femoral Neck System (FNS) and multiple cannulated screw (CS) fixation in patients with femoral neck fractures (FNFs) at a minimum follow-up of 24 months, as well as to identify factors associated with ONFH after FNS fixation. **Methods:** A retrospective cohort study was conducted at a single university hospital involving patients aged ≥18 years with isolated FNFs treated by closed reduction and internal fixation using either FNS or multiple CS. Reduction quality, fracture union, incidence and extent of ONFH (measured by Kerboul angle), and revision surgery due to ONFH or other causes were assessed as outcome measures. Logistic regression analyses were conducted to identify factors associated with ONFH after FNS fixation. **Results:** Eighty-seven patients were included, comprising 48 in the FNS group and 39 in the CS group. No significant differences were found between the groups in reduction quality, union rate, or time to union (*p* > 0.05 for all). The incidence of ONFH did not differ significantly between the FNS and CS groups (*p* = 0.314). However, the FNS group showed a significantly greater extent of ONFH (*p* = 0.031) and longer mean time to ONFH diagnosis (*p* = 0.001). No significant differences were observed in revision surgery rates due to ONFH (*p* = 1.000) or other causes (*p* = 0.624). Multivariate analysis identified fracture displacement (Garden classification stages III and IV) and poor reduction quality (“Broken S” by Lowell’s criteria) as significant predictors of ONFH after FNS fixation. **Conclusions:** ONFH following FNS fixation tended to exhibit a larger necrotic area and a delayed radiographic onset compared to CS fixation; however, the overall incidence and revision rates were comparable between implants. These differences may be due to initial fracture displacement and baseline severity, rather than the FNS implant itself. In the FNS group, the mean time to radiographic diagnosis of ONFH was 13.1 months, and initial fracture displacement and reduction quality were identified as risk factors for the development of ONFH following FNS fixation. Therefore, long-term and vigilant follow-up is essential after FNS fixation, particularly in patients with displaced fractures. Achieving optimal reduction quality and anatomical femoral neck alignment remains paramount to mitigating the risk of ONFH after FNS fixation.

## 1. Introduction

Femoral neck fracture (FNF) represents a major clinical challenge because of its high complication rate, substantial socioeconomic burden, and increasing incidence [1,2,3,4]. Internal fixation is an important treatment option aimed at preserving the native hip joint in patients with FNFs [5]. Multiple cannulated screw (CS) and dynamic hip screw (DHS) techniques are commonly used for internal fixation of FNFs. A recently developed novel implant—the Femoral Neck System (FNS; DePuy-Synthes, Johnson & Johnson Medical Devices, New Brunswick, NJ, USA)—has been widely adopted for its minimally invasive approach, fixed-angle stability, and improved rotational resistance [6,7,8,9].

Osteonecrosis of the femoral head (ONFH) is one of the most serious complications following FNFs, with reported incidences as high as 39.5% [10,11,12]. Progression of ONFH may result in femoral head collapse, causing severe pain, functional limitation, and secondary arthritis that often requires revision arthroplasty [12,13]. Although ONFH can occur shortly after injury, it is also known to develop insidiously, even more than one year post-injury [14,15]. A follow-up period of at least 24 months is recommended for accurate assessment [16].

Although biomechanical and short-term clinical studies have reported promising outcomes for FNS, long-term data on the incidence of ONFH after FNS fixation remain limited [7,8,15,17,18,19]. Clinical observations during routine follow-up suggest that ONFH after FNS fixation may involve a greater extent of necrosis and develop at a later period. Furthermore, although previous studies have identified fracture type and reduction quality as contributors to ONFH following CS or DHS fixation, evidence on specific factors associated with FNS fixation remains limited [14,20,21,22].

This study aimed to compare the incidence, extent, and time to diagnosis of ONFH between FNS and multiple CS fixation in patients with FNFs after at least 24 months of follow-up, as well as to identify factors associated with ONFH following FNS fixation.

## 2. Materials and Methods

### 2.1. Study Design and Patients

This retrospective study included patients who underwent internal fixation for FNF at a single university hospital between June 2014 and February 2023. This study was approved by the authors’ affiliated institutions. The need for informed consent was waived because of the retrospective nature of the study.

Inclusion criteria were patients aged ≥18 years, isolated FNF, surgery performed within 48 h of injury, and a minimum follow-up period of ≥24 months. Exclusion criteria included a follow-up period of <24 months, internal fixation using implants other than FNS or CS, concomitant fractures at other regions, or pathological fractures.

All patients diagnosed with FNF were offered two treatment options: internal fixation and arthroplasty. The advantages and disadvantages of each were explained, and the patient’s participation in the decision-making process was ensured. When patients desired joint preservation through internal fixation, the high risk of failure or complications based on fracture characteristics was explained, and internal fixation was performed with the patient’s consent. Conversely, when patients preferred arthroplasty, consultation with a hip surgeon was arranged to facilitate arthroplasty. Two different methods of internal fixation were used depending on the time period. Multiple CS fixation was performed from June 2014 to May 2019, and FNS fixation was performed from June 2019 to February 2023. During the study period, other fixation devices, such as DHS, were used only in patients who did not meet the inclusion criteria (e.g., pathological fractures or concomitant injuries) and were therefore excluded from the present cohort.

Patients were classified into two groups based on the implant used for internal fixation: FNS or CS. Demographics and clinical variables, including age, sex, body mass index (BMI), diabetes, smoking status, chronic kidney disease (CKD), American Society of Anesthesiologists classification, bone mineral density (BMD), osteoporosis, injury mechanism, follow-up period, and operative time, were collected through an analysis of electronic medical record analysis. BMD was assessed in patients previously diagnosed with osteoporosis or those aged ≥65 years. Injury mechanisms were classified as low-energy (ground-level fall or fall < 1 m) or high-energy (fall ≥ 1 m or traffic accident).

Fracture characteristics, including fracture location (subcapital, transcervical, or basicervical), Garden classification, and Pauwels classification, were determined using initial hip anteroposterior (AP) and lateral radiographs. Preoperative computed tomography (CT) was routinely performed for all patients to evaluate fracture comminution.

### 2.2. Outcome Measures

Radiographic outcomes included Garden alignment index, Lowell’s criteria, residual displacement, tip–apex distance (TAD), sliding distance, union, time to union, ONFH diagnosis, Kerboul angle, and the interval from injury to ONFH diagnosis. The Garden alignment index was evaluated on the immediate postoperative AP and lateral hip radiographs. On the AP radiograph, alignment was classified as good when the angle between the medial cortex of the femoral shaft and the central axis of the primary compressive trabeculae of the femoral head ranged from 160° to 175°, and poor when outside this range. On the lateral radiograph, alignment was defined as good when the angle between the primary compressive trabeculae and the femoral shaft ranged from 170° to 190°, and poor when outside this range. Lowell’s criteria were classified as “Intact S” when cortical continuity between the femoral head and neck formed a smooth S-shaped or inverted S-shaped curve on both AP and lateral radiographs. If cortical continuity appeared flat or angular on either radiograph, it was defined as a “Broken S”. Residual displacement was evaluated using CT scans, routinely performed immediately after surgery. Residual displacement was classified as good when postoperative displacement was <2 mm and poor when ≥2 mm. TAD was defined as the sum of the distances from the bolt tip to the femoral head apex on AP and lateral radiographs in the FNS group, and as the sum of the mean distances from the femoral head apex to the tip of each screw on AP and lateral radiographs in the CS group [8,23]. Sliding distance was defined as the distance from the femoral head apex to the lateral cortex, measured along a line drawn through the centers of the femoral head and neck on immediate postoperative and follow-up AP radiographs [24]. TAD and sliding distance measurements were adjusted for magnification using the bolt diameter of the FNS implant or the diameter of the CS [25]. Bone union was defined radiographically as the disappearance of the fracture line in at least three of the four cortices in AP and lateral radiographs. Nonunion was defined as failure to achieve radiographic union within six months postoperatively [26]. ONFH was diagnosed when the Steinberg classification stage ≥ 2 was observed on follow-up radiographs [27]. The Kerboul angle was used to quantify the extent of the osteonecrotic involvement. The angle was calculated as the sum of necrotic angles, measured from the femoral head center on AP and lateral radiographs [28]. Radiographic outcomes were independently assessed in a blinded manner by two orthopedic surgeons who were not involved in the surgical procedures. In cases of disagreement, a third independent orthopedic surgeon with fellowship training in orthopedic trauma was consulted to reach a consensus.

The clinical outcome was defined as the need for revision surgery. The reasons for revision were categorized as related to ONFH or other causes.

### 2.3. Surgical Technique

A single surgeon performed all surgeries. Each patient was positioned supine on a fracture table under general anesthesia, and closed reduction was performed under C-arm guidance. Open reduction was not required in any case. For FNS fixation, a guide pin was inserted into the central axis of the femoral head after closed reduction. After reaming to an appropriate depth, the bolt was attached to the lateral plate and inserted. An anti-rotation screw of the same length as the bolt was inserted, followed by placement of distal locking screws [8]. A two-hole plate was used in all cases. For multiple CS fixation, three parallel, partially threaded CSs (DePuy-Synthes, Johnson & Johnson Medical Devices, New Brunswick, NJ, USA) were inserted into the femoral head in an inverted triangular configuration, with screw tips placed 5–10 mm from the subchondral bone. The lateral cortical entry point of the inferior screw was located above the lesser trochanter in all cases [8].

### 2.4. Postoperative Rehabilitation and Follow-Up

All patients followed a standardized postoperative rehabilitation protocol. Active range-of-motion exercises were initiated immediately postoperatively. Partial weight-bearing (PWB) using walking aids was permitted once patients achieved 45° of active hip flexion. For the first 6 weeks following surgery, patients were instructed to use assistive devices (e.g., crutches or a walker) with strictly limited PWB of 15 kg on the affected limb. From 6 weeks postoperatively, the weight-bearing limit was increased to 30 kg. At 3 months post-surgery, patients were allowed to progress to full weight-bearing (FWB) and ambulate without assistive devices, as tolerated by their pain and physical condition. Patients who were unable to safely use assistive devices were mobilized in a wheelchair. Routine outpatient orthopedic follow-up visits were scheduled at 1, 2, 3, 6, 9, and 12 months postoperatively, and every 6 months thereafter. Plain hip radiographs were obtained at each visit.

### 2.5. Statistical Analysis

Comparative analyses were performed between the FNS and CS groups. Categorical variables were analyzed using the chi-square or Fisher’s exact tests. Continuous variables were tested for normality using the Shapiro–Wilk test. Normally distributed variables were analyzed using the independent *t*-test, and non-parametric data were analyzed with the Mann–Whitney U test. Interobserver reliability for the radiographic assessments (e.g., Garden classification, Kerboul angle) was evaluated using Cohen’s kappa coefficient or intraclass correlation coefficient (ICC). Factors associated with ONFH after FNS fixation were identified through univariate and multivariate logistic regression analyses. To prevent model overfitting and maintain model stability, only variables with *p* < 0.1 on univariate analysis were included in a multivariate logistic regression model using the forward stepwise method. Results of logistic regression are presented as odds ratios (ORs) with 95% confidence intervals (CIs). All statistical analyses were performed using IBM SPSS Statistics, Version 27.0 (IBM Corp., Armonk, NY, USA), with statistical significance set at *p* < 0.05.

## 3. Results

### 3.1. Study Population

**Figure 1:**
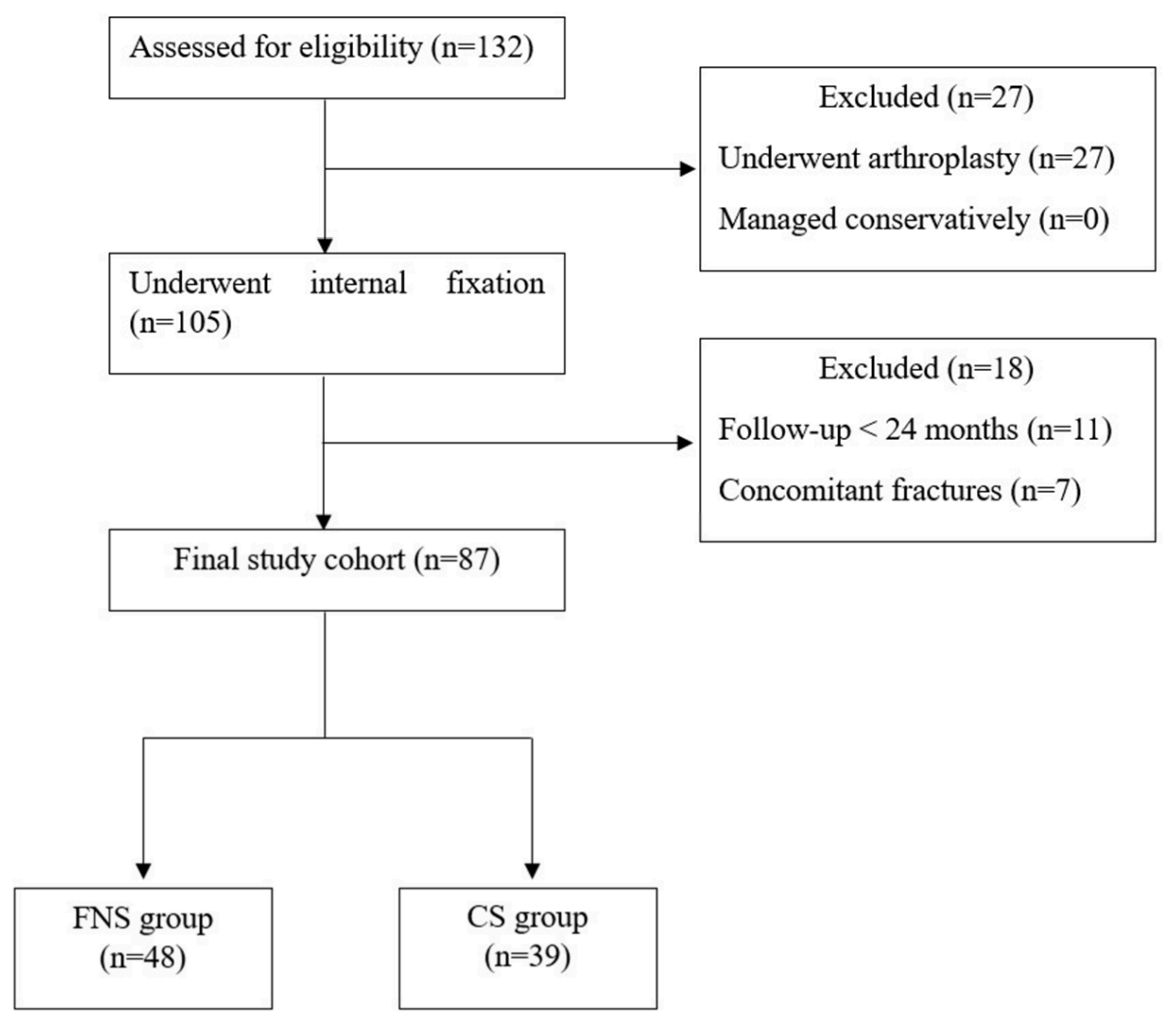
Patient selection flow diagram.

During the study period, 132 patients with femoral neck fractures were identified. Based on the inclusion and exclusion criteria, a total of 87 patients were ultimately included in the final analysis; of these, 48 were in the FNS group and 39 were in the CS group (Figure 1).

### 3.2. Patient Characteristics and Outcomes

The mean age of the cohort was 55.9 ± 10.1 years (range: 30–84 years). Eleven patients had diabetes, 27 were current smokers, and six had CKD. BMD was measured in 57 patients, with a mean T-score of −2.5 ± 0.9 (range: −0.9 to −4.7); osteoporosis was diagnosed in 31 of them. The mean follow-up period was 34.6 ± 19.1 months (range: 24–120 months). Fracture locations were predominantly at the subcapital region (50 patients, 57.5%), followed by transcervical (33 patients, 37.9%) and basicervical regions (4 patients, 4.6%). Displaced fractures classified as Garden stages III and IV were identified in 35 patients (40.2%), and Pauwels Type III fractures in 18 patients (27.0%). Among the 11 patients lost to follow-up, five had received FNS fixation and six had received CS fixation. The mean follow-up period was 10.1 ± 3.6 months (range: 6–18 months). No cases of ONFH or evidence of failure were observed during the follow-up period in these patients.

The FNS group (mean age, 56.6 years; 19 men, 29 women) and the CS group (mean age, 55.9 years; 17 men, 22 women) showed statistically similar demographics and fracture characteristics (*p* > 0.05 for all). Only the operative time was significantly shorter in the FNS group (*p* = 0.027) (Table 1).

No significant differences were found between groups in Garden alignment indices (AP and lateral), Lowell’s criteria, or residual displacement (*p* > 0.05 for all) (Table 2). The mean TAD (FNS group: 17.7 ± 4.8 mm, CS group: 16.1 ± 4.4 mm, *p* = 0.122) and sliding distance (FNS group: 2.8 ± 4.0 mm, CS group: 2.3 ± 3.7 mm, *p* = 0.340) were comparable. Bone union was achieved in 46 of 48 patients (95.8%) in the FNS group and 38 of 39 patients (97.4%) in the CS group, with no significant difference between groups (*p* = 1.000). The mean time to union did not differ significantly between groups (FNS group: 4.8 ± 1.2 months, CS group: 4.4 ± 1.3 months, *p* = 0.391) (Table 2). The inter-observer reliability for the radiographic measurements demonstrated excellent agreement. The kappa values were 0.836 for the Garden classification, 0.810 for the Pauwels classification, 0.854 for Lowell’s criteria, and 1.000 (AP) and 0.851 (lateral) for the Garden alignment index. For the Kerboul angle, the ICC was 0.948 for the AP view and 0.912 for the lateral view.

**Figure 2:**
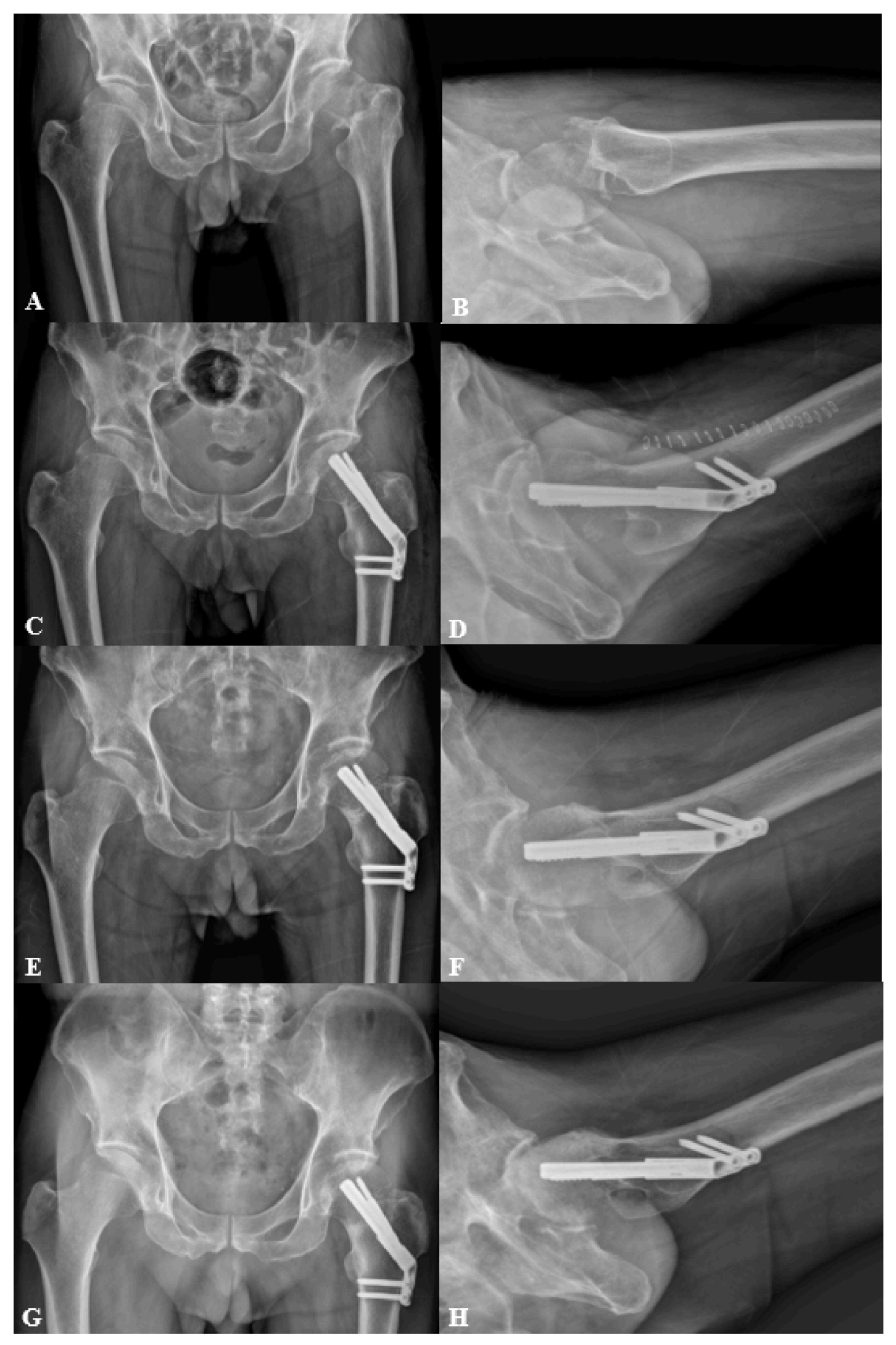
A 61-year-old male sustained a left femoral neck fracture (FNF) following a ground-level fall. Initial anteroposterior (**A**) and lateral (**B**) radiographs show a displaced subcapital fracture, classified as Garden stage IV. Closed reduction and internal fixation using the Femoral Neck System (FNS) was performed on the day of injury. Immediate postoperative anteroposterior (**C**) and lateral (**D**) radiographs demonstrate satisfactory reduction and implant placement. At six months postoperatively, anteroposterior (**E**) and lateral (**F**) radiographs confirm fracture union. Although the patient remained asymptomatic, anteroposterior (**G**) and lateral (**H**) radiographs obtained at 18 months postoperatively show subchondral sclerosis and femoral head flattening, consistent with osteonecrosis of the femoral head (ONFH). As the patient remained functional and symptom-free, no revision surgery was performed, and the condition was managed conservatively.

During follow-up, ONFH was diagnosed in 14 patients (29.2%) in the FNS group and seven patients (17.9%) in the CS group, with no significant difference between groups (*p* = 0.314). The mean total Kerboul angle was significantly greater in the FNS group (147.5 ± 62.8°; median, 121.0°; interquartile range (IQR), 98.3–204.3°) than in the CS group (106.9 ± 14.7°; median, 113.0°; IQR, 100.0–117.0°) (*p* = 0.036). The AP Kerboul angle was also greater in the FNS group (70.4 ± 31.7°; median, 62.0°; IQR, 49.0–101.3°) than in the CS group (49.3 ± 7.3°; median, 49.0°; IQR, 45.0–54.0°) (*p* = 0.031), whereas the lateral angle showed no significant difference (*p* = 0.080) (Table 2). However, a subgroup analysis limited to undisplaced fractures (Garden stages I and II) revealed no statistically significant differences between the two groups across any parameter: AP (FNS: 57.0 ± 39.8° vs. CS: 48.8 ± 3.6°, *p* = 0.756), lateral (FNS: 87.0 ± 63.8° vs. CS: 62.2 ± 5.6°, *p* = 0.571), or total Kerboul angles (FNS: 144.0 ± 103.6° vs. CS: 111.0 ± 8.8°, *p* = 0.637). The mean time to ONFH diagnosis was significantly longer in the FNS group (13.1 months) than in the CS group (5.7 months) (*p* = 0.001). Notably, seven patients (14.6%) in the FNS group were diagnosed with ONFH within 12 months, and an equal number were diagnosed after 12 months. In contrast, all ONFH cases in the CS group were diagnosed within the first 12 months (*p* = 0.047) (Table 2). Figure 2 shows a case of ONFH that developed 18 months after FNS fixation. As the patient remained functional and symptom-free, no revision surgery was performed, and the condition was managed conservatively.

**Figure 3:**
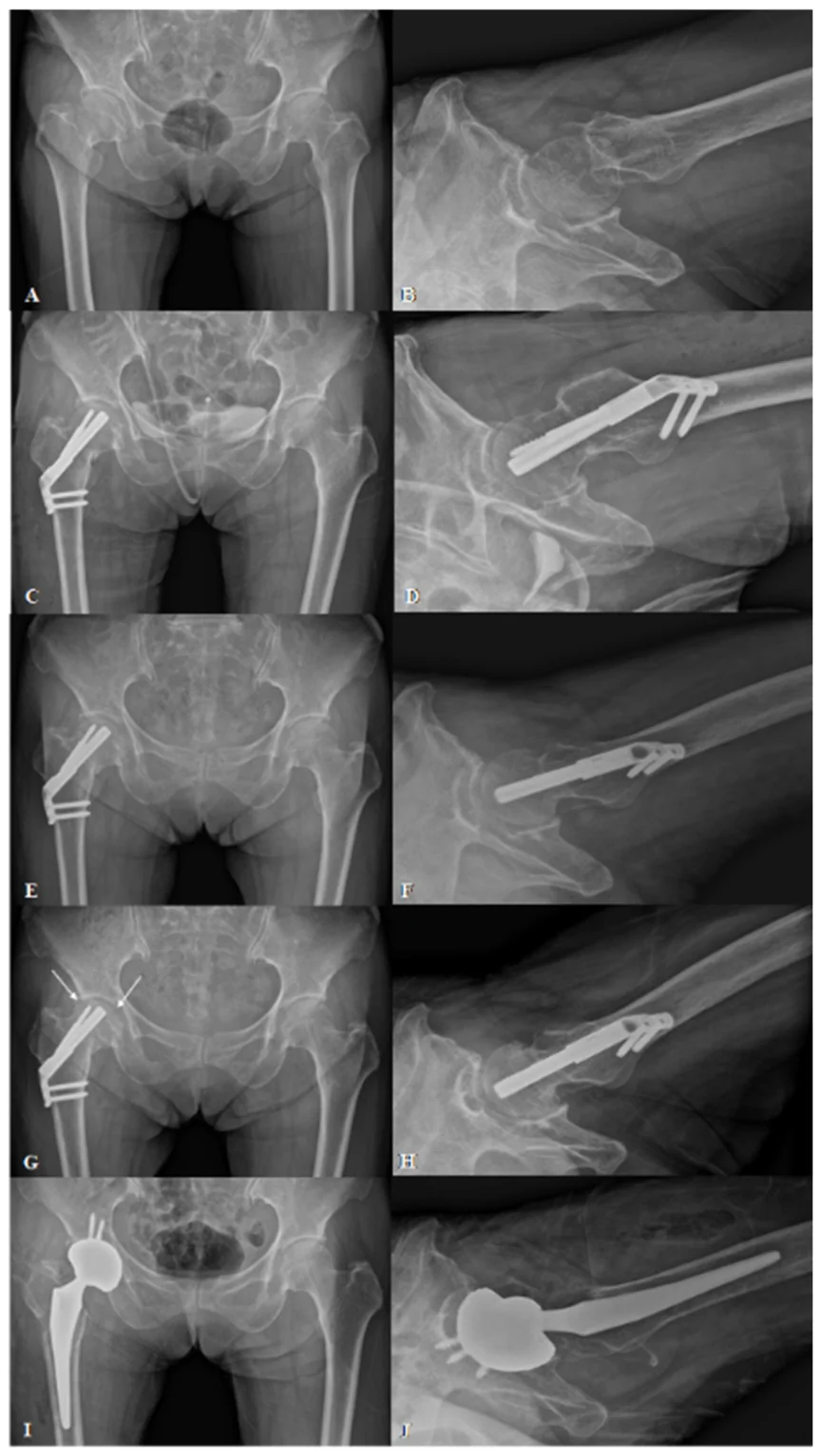
A 63-year-old female sustained a right femoral neck fracture following a ground-level fall. Initial anteroposterior (**A**) and lateral (**B**) radiographs show a displaced, complete subcapital fracture. Closed reduction and internal fixation with the Femoral Neck System (FNS) was performed on the day of injury. Immediate postoperative anteroposterior (**C**) and lateral (**D**) radiographs demonstrate satisfactory reduction and proper implant placement. At six months postoperatively, anteroposterior (**E**) and lateral (**F**) radiographs show radiographic evidence of fracture union. At 10 months postoperatively, the patient developed right hip pain. Anteroposterior radiograph (**G**) reveals femoral head collapse and flattening (white arrows), indicating osteonecrosis of the femoral head (ONFH), while the lateral radiograph (**H**) shows relatively less collapse. Due to progressive symptoms limiting daily activities, the patient underwent revision surgery. Anteroposterior (**I**) and lateral (**J**) radiographs after revision with total hip arthroplasty.

Revision surgery for ONFH was performed in five of 14 patients (35.7%) in the FNS group and three of seven patients (42.9%) in the CS group, with no significant difference between the groups (*p* = 1.000). Revision surgeries for other reasons included two cases of nonunion and one case of excessive sliding in the FNS group, and one case of nonunion in the CS group. Revisions for causes other than ONFH (*p* = 0.624) and total revision rate (*p* = 0.535) did not differ significantly between the groups (Table 2). Figure 3 shows a case of ONFH with femoral head collapse that developed 10 months after FNS fixation. Due to progressive symptoms limiting daily activities, the patient underwent revision surgery. Detailed information on patients who developed ONFH is presented in Table 3.

In the univariate logistic regression analysis, displaced fractures (Garden classifications III and IV) were significantly associated with ONFH after FNS fixation (OR = 6.72, 95% CI: 1.57–28.88, *p* = 0.010). Additionally, Lowell’s criteria (“Broken S”) showed a significance level of *p* < 0.1 and was therefore included in the multivariate analysis (Broken S, OR = 4.17, 95% CI: 0.92–18.88, *p* = 0.064) (Table 4). The multivariate logistic regression analysis indicated that displaced fracture (Garden III and IV) (OR = 9.61, 95% CI: 1.78–52.08, *p* = 0.009) and the “Broken S” pattern according to Lowell’s criteria (OR = 7.18, 95% CI: 1.10–46.97, *p* = 0.040) were significantly associated with ONFH after FNS fixation (Table 4).

## 4. Discussion

This study investigated the incidence, extent, and time to diagnosis of ONFH following fixation with the FNS and multiple CS in FNFs with a minimum follow-up of 24 months. The findings demonstrated no significant difference in the incidence of ONFH between the FNS and CS groups. However, ONFH in the FNS group tended to involve a larger necrotic area, although this did not result in a higher revision rate. Additionally, ONFH occurred later in the FNS group, and its development was associated with initial fracture displacement and the quality of reduction.

In this study, the extent of ONFH, measured by the Kerboul angle, was significantly greater in the FNS group. This finding may be partially attributable to the higher proportion of displaced fractures (Garden stages III and IV) in the FNS group, as fracture displacement correlates with vascular disruption. Recent dynamic contrast-enhanced MRI and CT-angiography studies, as well as earlier scintigraphy studies, have shown that the superior retinacular artery is vulnerable to disruption during FNFs because it adheres closely to the femoral neck, and that the inferior retinacular artery and intraosseous epiphyseal anastomosis play crucial roles in maintaining femoral head perfusion [29,30,31,32]. Previous randomized trials comparing DHS fixation with multiple CS fixation have shown a higher incidence of ONFH with DHS, and one study attributed this finding to the reamer design of DHS [20,33]. Although direct evidence is not available in our cohort, based on previous study results, it can be cautiously inferred that reaming during FNS bolt insertion may cause iatrogenic injury to the intraosseous epiphyseal network, thereby reducing microvascular perfusion of the femoral head [29,30,31,32]. However, it must be emphasized that concerns regarding the impact of reaming on the intraosseous vascular network remain entirely hypothetical. Because our study lacks direct histological, angiographic, or dynamic contrast-enhanced MRI evidence, clinical studies utilizing advanced imaging modalities are required to establish whether the FNS reamer design is associated with an increased extent of ONFH. Although ONFH was more extensive in the FNS group, revision rates did not differ, suggesting that lesion size alone may not directly indicate the severity of femoral head collapse. Prospective studies are needed to clarify the interplay among lesion size, reamer effects, and clinical outcomes.

In this study, ONFH was diagnosed an average of 13 months following FNS fixation. A recent study with a mean follow-up of 18 months after FNS fixation reported a comparable onset time of 19 months [15], consistent with our results. This delayed onset may be attributable to the vascular effects of the FNS implant. FNFs often disrupt the blood supply to the femoral head; however, adequate stabilization can promote revascularization via ingrowth of vascular buds [34,35]. During revascularization, blood flow is redirected through collateral anastomotic networks [29,30]. Recent micro-CT and angiographic studies suggest that multiple CS fixation configured in an inverted triangle may injure major branches of the epiphyseal anastomosis, significantly impairing femoral head perfusion [30,31]. In contrast, central fixation techniques, such as FNS, are thought to preserve the epiphyseal anastomotic vasculature better [30,31]. Furthermore, although previous observational studies have reported no significant association between TAD and revision surgery after FNS fixation, they emphasized that the central bolt placement is critical for favorable outcomes [19,36]. Integrating these literature findings, we cautiously speculate that ONFH might manifest more rapidly following CS fixation due to potential mechanical compromise of major peripheral anastomotic branches. Nevertheless, these proposed vascular injury patterns (central vs. peripheral) represent indirect clinical reasoning, rather than proven anatomical phenomena. Ultimately, successful FNF fixation likely requires preservation of the intraosseous anastomotic network and the attainment of substantial biomechanical stability.

This study identified fracture displacement (Garden classification III and IV) and reduction quality (Lowell’s “broken S”) as significant predictors of ONFH after FNS fixation. However, because the multivariable logistic regression analysis was based on a relatively small number of ONFH events (*n* = 14), these identified predictors should be interpreted with caution. This limited event rate accounts for the wide confidence intervals observed in the odds ratios and may limit precision when evaluating the exact statistical magnitudes of these risk factors. Nevertheless, considering that the extensive literature on internal fixation of femoral neck fractures has consistently demonstrated fracture displacement and inadequate reduction quality to be major risk factors for ONFH [14,37,38], it is highly plausible that these variables remain critical determinants of ONFH development following FNS fixation. Fracture displacement directly correlates with the severity of vascular disruption, while inadequate fracture reduction could cause abnormal biomechanical stresses that impair femoral head revascularization [34,39]. Therefore, achieving anatomical reduction and restoring normal femoral neck alignment remains essential for minimizing the risk of ONFH.

The observed ONFH incidence of 29% at a minimum follow-up of 24 months after FNS fixation appears higher than those reported in studies with shorter follow-up periods. One study with a mean follow-up period of seven months after FNS fixation reported an ONFH incidence of 4.9%, whereas another with a one-year follow-up reported an incidence of 11.8% [19,36]. In this study, ONFH occurred in 4.2% of cases within seven months and in 14.6% within 12 months, a finding that is comparable to previous reports. This finding indicates that short-term studies may underestimate the true incidence of ONFH. According to a recent 10-year follow-up study of 160 patients who underwent internal fixation following FNFs, the average time to conversion to arthroplasty due to ONFH was 22.2 months [16]. Considering that conversion to arthroplasty is typically performed when ONFH has progressed to a severe, symptomatic stage, the minimum follow-up period of 24 months in this study—as well as the mean follow-up periods of 30.0 months and 40.3 months for each group—is believed to have been sufficient to detect both early-stage and late-stage ONFH. Although there was a difference of approximately 10 months in the mean follow-up periods between the two groups, previous reports indicate that the vast majority of ONFH cases following FNFs occur within 12–24 months after injury. Therefore, the risk of missing new-onset ONFH cases in the FNS group due to this 10-month discrepancy beyond the 24-month minimum is likely to be minimal. Because this difference in follow-up duration was not statistically significant, its confounding impact on cumulative ONFH incidence is considered limited. Additionally, considering that the average time to ONFH diagnosis in the FNS group was 13.1 months in this study, a follow-up period of at least 24 months appears necessary. Although many cases of ONFH may remain asymptomatic, it is important to explain the current status to patients when ONFH is observed during follow-up. Informing them that symptoms may develop later and that additional surgery might be necessary is crucial for enhancing patient compliance and preventing potential litigation. There may be a potential increase in healthcare costs associated with long-term follow-up. However, as demonstrated in this study, where only plain hip radiographs at 6-month intervals are required, the cost increase is likely minimal. In summary, long-term and vigilant follow-up extending beyond 24 months after FNS fixation may be warranted, particularly for displaced fractures (Garden stages III and IV). Furthermore, surgeons should be mindful that achieving optimal reduction quality and restoring anatomical femoral neck alignment remain essential for minimizing the risk of ONFH after FNS fixation.

This study’s limitations include its study design, relatively small sample size, low incidence of certain variables (e.g., poor Garden alignment), inability to quantify ONFH lesion volume, and lack of patient-reported outcome measures. Furthermore, given the retrospective design, intraoperative visual data (e.g., intraoperative photographs or detailed fluoroscopic records) that would allow for direct confirmation of subtle technical differences were unavailable. To compensate for this, we utilized objective radiographic indicators such as the Kerboul angle. However, other than plain radiographs, routine hip magnetic resonance imaging (MRI) was not performed on all patients. Relying solely on plain radiographs may have led to the underestimation or delayed identification of ONFH. Because MRI is substantially more sensitive for detecting early, subclinical ONFH before it manifests radiographically, the delayed onset observed in the FNS group might partially reflect a lag in radiographic detection, rather than a purely biological delay. Therefore, future prospective studies utilizing advanced imaging modalities, such as MRI or single-photon emission computed tomography (SPECT/CT), are warranted to precisely determine the true onset timeline of ONFH. Furthermore, this study was conducted at a single institution involving a homogeneous Korean population. Therefore, to firmly establish the generalizability of these findings, future multicenter studies incorporating multi-ethnic cohorts are required. Although there were no statistically significant differences in fracture characteristics between the two groups, potential selection bias exists. The proportion of displaced fractures (Garden stages III and IV) and the rate of comminution were higher in the FNS group, which may have contributed to the greater extent of ONFH observed in this group. Additionally, the non-randomized, sequential cohort design—using the two implants during different time periods—is another major limitation, as this sequential allocation introduces potential chronological confounders, such as the evolution of the surgeon’s surgical proficiency due to the learning curve, variations in perioperative management, and modifications to rehabilitation protocols. However, institutional perioperative care protocols remained strictly consistent throughout the entire study duration, with minor alterations limited to wound dressing materials. Furthermore, standardized postoperative rehabilitation protocols—specifically regarding the initiation timing and progressive intensity of partial weight-bearing—were applied identically across both cohorts, and are thus unlikely to have introduced significant confounding bias. Finally, regarding the surgical learning curve associated with adopting the FNS, its insertion instrumentation and trajectory alignment share strong technical similarities with the conventional dynamic hip screw (DHS) system, which the single operating surgeon was already highly proficient in. Therefore, any confounding effect stemming from a technical learning curve is believed to be negligible. Nevertheless, given the historical nature of sequential retrospective cohorts, unmeasured temporal confounders and shifting patient-selection patterns over time may have introduced clinical bias and influenced the study outcomes (e.g., size of ONFH). Therefore, well-designed prospective randomized trials are warranted to minimize such temporal biases.

This study’s strengths include a minimum two-year follow-up for accurate ONFH detection, a consistent surgical technique performed by a single surgeon, and standardized rehabilitation protocols, which enhance the reliability and validity of the findings. Future prospective, multicenter studies with advanced imaging techniques are required to identify ONFH lesions accurately.

## 5. Conclusions

ONFH following FNS fixation tended to exhibit a larger necrotic area and a delayed radiographic onset compared to CS fixation; however, the overall incidence and revision rates were comparable between the two implants. Given the limitations of this study—specifically the historical comparison between the two treatment periods, unequal follow-up durations, radiograph-based detection of ONFH, single-center design, and limited number of ONFH cases—these differences may be due to initial fracture displacement and baseline severity, rather than the FNS implant itself. In the FNS group, the mean time to radiographic diagnosis of ONFH was 13.1 months, and initial fracture displacement (Garden classification stages III and IV) and reduction quality (Lowell’s “broken S”) were identified as risk factors for the development of ONFH following FNS fixation. Therefore, long-term and vigilant clinical follow-up is essential after FNS fixation, particularly in patients with displaced fractures. Achieving optimal reduction quality and anatomical femoral neck alignment remains paramount to mitigating the risk of ONFH after FNS fixation.

## Figures and Tables

**Table 1:** Baseline demographics and fracture characteristics of the patients.

	FNS Group (*n* = 48)	CS Group (*n* = 39)	*p*-Value
Age	56.6 ± 8.4	55.9 ± 15.0	0.259
Sex (%)			0.827
Male	19 (39.6)	17 (43.6)	
Female	29 (60.4)	22 (56.4)	
BMI (kg/m^2^)	22.6 ± 3.5	22.1 ± 3.0	0.542
DM (%)	4 (8.3)	7 (17.9)	0.209
Smoking (%)	12 (25.0)	14 (35.9)	0.347
CKD (%)	2 (4.2)	4 (10.3)	0.401
ASA classification (%)			0.207
1	21 (43.8)	10 (25.6)	
2	21 (43.8)	24 (61.5)	
3	6 (12.4)	5 (12.9)	
BMD (T-score)	−2.5 ± 0.8	−2.6 ± 0.9	0.719
Osteoporosis (%)	19/33 (57.6)	12/24 (50.0)	0.601
Injury mechanism (%)			0.666
Low	27 (56.3)	24 (61.5)	
High	21 (43.7)	15 (38.5)	
Follow-up (month)	30.0 ± 10.6	40.3 ± 25.1	0.151
Operative time (min)	52.3 ± 11.9	57.7 ± 10.5	**0.027**
Fracture location (%)			0.112
Subcapital	29 (60.4)	21 (53.8)	
Transcervical	15 (31.3)	18 (46.2)	
Basicervical	4 (8.3)	0 (0)	
Garden classification (%)			0.501
Stage I	20 (41.7)	17 (43.6)	
Stage II	5 (10.4)	8 (20.5)	
Stage III	15 (31.3)	10 (25.6)	
Stage IV	8 (16.7)	4 (10.3)	
Displacement (%)			0.284
Non-displaced (Garden Stages I & II)	25 (52.1)	25 (64.1)	
Displaced (Garden Stages III & IV)	23 (47.9)	14 (35.9)	
Pauwels classification (%)			0.196
Type I	9 (18.8)	14 (35.9)	
Type II	28 (58.3)	18 (46.2)	
Type III	11 (22.9)	7 (17.9)	
Comminuted fragment (%)			0.169
Present	18 (37.5)	9 (23.1)	
Absent	30 (62.5)	30 (76.9)	

BMI: body mass index, DM: diabetes mellitus, CKD: chronic kidney disease, ASA: American Society of Anesthesiologist, BMD: bone mineral density. Significant *p* values are highlighted in bold.

**Table 2:** Radiographic and clinical outcomes of the patients.

	FNS Group (*n* = 48)	CS Group (*n* = 39)	*p*-Value
Garden alignment index—AP			0.448
Good (160–175°)	48 (100)	38 (97.4)	
Poor	0 (0)	1 (2.6)	
Garden alignment index—Lateral			0.585
Good (170–190°)	47 (97.9)	37 (94.9)	
Poor	1 (2.1)	2 (5.1)	
Lowell’s criteria			1.000
Intact S	39 (81.3)	31 (79.5)	
Broken S	9 (18.7)	8 (20.5)	
CT residual displacement (%)			0.507
Good (<2 mm)	44 (91.7)	34 (87.2)	
Poor (≥2 mm)	4 (8.3)	5 (12.8)	
TAD (mm)	17.7 ± 4.8	16.1 ± 4.4	0.122
Sliding distance (mm)	2.8 ± 4.0	2.3 ± 3.7	0.340
Union (%)	46 (95.8)	38 (97.4)	1.000
Time to union (Month)	4.8 ± 1.2	4.4 ± 1.3	0.391
ONFH (%)	14 (29.2)	7 (17.9)	0.314
Kerboul angle (°)			
AP	70.4 ± 31.7 ^a^ 62.0 (49.0–101.3) ^b^	49.3 ± 7.3 ^a^ 49.0 (45.0–54.0) ^b^	**0.031**
Lateral	77.1 ± 36.6 ^a^ 71.5 (43.5–107.0) ^b^	57.6 ± 10.0 ^a^ 58.0 (53.0–67.0) ^b^	0.080
Total	147.5 ± 62.8 ^a^ 121.0 (98.3–204.3) ^b^	106.9 ± 14.7 ^a^ 113.0 (100.0–117.0) ^b^	**0.036**
Time to ONFH diagnosis (Month)	13.1 ± 6.2	5.7 ± 1.0	**0.001**
Time interval to ONFH (%)			**0.047**
<12 months	7 (14.6)	7 (17.9)	
≥12 months	7 (14.6)	0 (0)	
Revision surgery (%)			
After ONFH	5/14 (35.7)	3/7 (42.9)	1.000
Other cause	3 (6.3)	1 (2.6)	0.624
Overall	8 (16.7)	4 (10.3)	0.535

CT: computed tomography, TAD: tip–apex distance, ONFH: osteonecrosis of femoral head. Significant *p* values are highlighted in bold. ^a^ mean ± standard deviation. ^b^ median values (interquartile ranges).

**Table 3:** Information regarding patients with ONFH.

No	Group	Age (Year)	Sex	Injury Mechanism	F/U (Months)	Fracture Location	Garden Classification	Pauwels Classification	Garden Alignment (AP/Lateral)	Lowell’s Criteria	TAD (mm)	Time to ONFH (Month)	Kerboul Angle (°)	Revision Surgery
1	FNS	57	Female	Low	58	Subcapital	I	II	Good/Good	Broken	26.5	21	58	No
2	FNS	34	Male	High	36	Transcervical	III	II	Good/Good	Intact	16.5	23	103	No
3	FNS	52	Male	High	24	Subcapital	III	II	Good/Good	Intact	19.5	18	99	No
4	FNS	59	Female	Low	26	Subcapital	IV	II	Good/Good	Broken	18.0	10	211	Yes
5	FNS	61	Male	Low	25	Transcervical	III	III	Good/Good	Broken	23.5	18	190	No
6	FNS	62	Female	Low	58	Subcapital	III	II	Good/Good	Broken	26.5	18	106	No
7	FNS	60	Male	Low	24	Subcapital	II	II	Good/Good	Intact	28.0	9	259	Yes
8	FNS	39	Male	High	56	Transcervical	III	II	Good/Good	Intact	18.6	14	162	Yes
9	FNS	48	Female	High	36	Subcapital	III	II	Good/Good	Intact	17.3	18	95	No
10	FNS	54	Female	Low	32	Subcapital	IV	III	Good/Good	Intact	23.5	8	202	Yes
11	FNS	60	Female	Low	36	Subcapital	III	II	Good/Good	Broken	19.6	4	96	No
12	FNS	84	Female	Low	28	Subcapital	I	II	Good/Good	Intact	20.3	5	115	No
13	FNS	64	Male	High	24	Subcapital	IV	III	Good/Good	Intact	13.6	8	127	No
14	FNS	63	Female	Low	24	Subcapital	IV	II	Good/Good	Intact	12.9	9	242	Yes
15	CS	53	Male	Low	94	Transcervical	II	II	Good/Good	Intact	9.0	6	100	Yes
16	CS	63	Female	High	47	Transcervical	IV	II	Good/Good	Intact	13.9	5	78	Yes
17	CS	66	Female	Low	24	Subcapital	II	I	Good/Good	Intact	25.1	6	117	Yes
18	CS	43	Female	High	24	Subcapital	I	I	Poor/Good	Broken	18.0	4	113	No
19	CS	58	Female	High	58	Subcapital	III	III	Good/Good	Broken	17.4	6	115	No
20	CS	53	Male	High	24	Subcapital	I	I	Good/Good	Intact	11.8	7	121	No
21	CS	56	Female	Low	38	Transcervical	I	II	Good/Good	Intact	12.7	6	104	No

ONFH: osteonecrosis of femoral head, F/U: follow-up, TAD: tip–apex distance, FNS: Femoral Neck System, CS: cannulated screw.

**Table 4:** Univariate and multivariate analysis of variables associated with the occurrence of ONFH after FNS fixation for femoral neck fractures (*n* = 48).

Variable	Univariate Analysis		Multivariate Analysis	
	Odds Ratio (95% CI)	*p*	Odds Ratio (95% CI)	*p*
Age (year)	1.01 (0.93–1.09)	0.862		
Female sex	0.83 (0.23–2.92)	0.766		
BMI (kg/m^2^)	0.94 (0.77–1.13)	0.497		
DM	0.80 (0.08–8.34)	0.848		
Smoking	2.14 (0.54–8.46)	0.277		
CKD	2.54 (0.15–43.7)	0.521		
ASA ≥ 3	0.45 (0.05–4.21)	0.481		
High-energy injury	0.63 (0.17–2.26)	0.473		
Osteoporosis	2.77 (0.47–16.46)	0.263		
Garden classification				
Non-displaced (Stage I, II)	Reference	—		
Displaced (Stage III, IV)	6.72 (1.57–28.88)	0.010 *	9.61 (1.78–52.08)	**0.009**
Pauwels classification				
Type I, II	Reference	—		
Type III	0.89 (0.69–7.30)	0.177		
Comminuted fragment				
Present	2.09 (0.59–7.45)	0.255		
Lowell’s criteria				
Broken S	4.17 (0.92–18.88)	0.064 *	7.18 (1.10–46.97)	**0.040**
Residual displacement				
<2 mm	Reference	—		
≥2 mm	1.25 (0.20–7.75)	0.811		
TAD				
<25 mm	Reference	—		
≥25 mm	3.00 (0.31–28.84)	0.341		
Sliding distance (mm)	0.98 (0.87–1.10)	0.767		
Operation time (min)	1.00 (0.95–1.05)	0.955		

ONFH: osteonecrosis of femoral head, FNS: Femoral Neck System (DePuy-Synthes), CI: confidence interval, BMI: body mass index, DM: diabetes mellitus, CKD: chronic kidney disease, ASA: American Society of Anesthesiologists, TAD: tip–apex distance. * Variables showing *p* < 0.1 in univariate analysis were included in the multivariate analysis model. Significant *p* values are highlighted in bold.

## Data Availability

The original contributions presented in this study are included in the article/Supplementary Materials. Further inquiries can be directed to the corresponding author.

## Supplementary Materials

The following supporting information can be downloaded at: https://www.mdpi.com/article/10.3390/jcm15166405/s1, Table S1: Raw data of demographics, clinical characteristics, clinical and radiographic outcomes for the entire study population.
